# Prevalence and factors of urinary incontinence among postpartum: systematic review and meta-analysis

**DOI:** 10.1186/s12884-023-06059-6

**Published:** 2023-10-28

**Authors:** Sidi Dai, Huating Chen, Taizhen Luo

**Affiliations:** 1 The Third Clinical College of Guangzhou Medical University, The Nursing College of Guangzhou Medical University, Guangzhou, China; 2 Department of Nursing, Guangdong Provincial Key Laboratory of Major Obstetric Diseases; Guangdong Provincial Clinical Research Center for Obstetrics and Gynecology; The Third Affiliated Hospital of Guangzhou Medical University, Guangzhou, China

**Keywords:** Meta-analysis, Urinary incontinence, Postpartum women, Risk factor

## Abstract

**Background:**

Postpartum urinary incontinence substantially impacts the psychophysical well-being of women. The influencing factors contributing to postpartum urinary incontinence remain a subject of contention in clinical investigation. By elucidating the factors contributing to postpartum urinary incontinence, more efficacious interventions for laboring women can be devised. Consequently, this review endeavored to scrutinize the repercussions of maternal postpartum urinary incontinence to furnish empirical references for the clinical advancement of preventive strategies.

**Method:**

The investigation employed bibliographic databases: Embase, PubMed, Web of Science, Cochrane Library, CBM, VIP, CNKI, and Wan Fang Data for article retrieval. A comprehensive consideration of all study designs was undertaken during the examination of the effects of postpartum urinary incontinence. The temporal limitation was set at all articles prior to February 2023. Studies incorporated laboring mothers experiencing normative labor and parturition. A total of 28,303 women were encompassed in the reviewed investigations.

**Results:**

A total of 5,915 putative citations were identified, from which 32 articles were selected for evaluating the effects of postpartum urinary incontinence. Meta-analyses revealed that the incidence of postpartum urinary incontinence was 26% [95%CI: (21% ~ 30%)]. Twelve pivotal variables were identified to influence postpartum urinary incontinence: cesarean delivery, vaginal delivery, age ≥ 35 years, multiparty (number of deliveries ≥ 2), neonatal weight > 4 kg, perineal dystonia, antecedents of urological incontinence-related pathology, maternal pre-conception BMI ≥ 24 kg/m^2, perineal laceration, instrumental parturition, historical pelvic surgical procedures, and protracted second stage of labor. Among these, cesarean delivery was identified as a protective factor against postpartum urinary incontinence.

**Conclusion:**

The study corroborated that anamnestic factors pertinent to urinary incontinence, vaginal parturitions, and neonates with a weight exceeding 4 kg serve as significant risk factors for postpartum urinary incontinence. Cesarean delivery emerged as a protective factor against postpartum urinary incontinence. Based on the prevalence of postpartum urinary incontinence, proactive intervention is requisite to mitigate the risk of postpartum urinary incontinence in postpartum women possessing these risk factors.

**Trial registration:**

CRD42023412096.

## Background

Urinary Incontinence constitutes a prevalent form of female pelvic floor dysfunction disorder. Postpartum Urinary Incontinence (PPUI) is correlated with biomechanical and endocrinological alterations that transpire during the obstetric phase and is attributable to perineal trauma sustained during parturition [1]. The International Continence Society (ICS) characterizes urinary incontinence as “an objectively verifiable, involuntary urinary dysfunction” [2], with stress urinary incontinence identified as the predominant subtype [3]. Parturition stands as a critical life event for women, and urinary incontinence profoundly compromises their psychophysical well-being and quality of life. Various studies have demonstrated that urinary incontinence augments the risk of postpartum depression [4–6]. Consequently, heightened vigilance towards the incidence of postpartum incontinence in women is imperative. At present, domestic and international investigations have probed the variables influencing postpartum incontinence, encompassing diverse modes of delivery, age, and parity [7, 8]. However, the caliber of these studies is heterogeneous, and the insights regarding the influencing factors are disparate. Research conducted in China indicates that age, gestational duration, and BMI do not constitute factors in the etiology of PPUI [9], whereas studies from other geographical locales suggest that gestational duration and BMI act as contributory factors in the development of PPUI [10]. In addition, variations exist in the statistical methodologies employed in disparate studies. Certain investigations [11] have employed multifactorial analyses superimposed upon unifactorial methods, while other studies [12] have solely relied on unifactorial analyses. The prevalence of PPUI ranges from 10 to 63% across distinct regions [13]. It is evident that the prevalence of PPUI varies inter-regionally, largely attributed to homogeneous medical standards within disparate regions and differential attention accorded to PPUI. To our knowledge, in extant reviews, scholars have concentrated on obstetric-related variables that contribute to the genesis of PPUI [14]. While gestation and parturition are principal causes of PPUI, non-obstetric variables warrant consideration as well. Accordingly, this study endeavored to explore the factors influencing maternal postpartum urinary incontinence and its prevalence via meta-analysis to furnish empirical references for the prophylaxis of maternal PPUI.

This study predominantly utilized meta-analysis, a quantitative technique employed to synthesize and juxtapose the outcomes of studies addressing analogous scientific queries. The veracity of the conclusions is contingent upon the quality of the incorporated studies, and it is frequently utilized for quantitative amalgamation in systematic reviews. By assimilating all pertinent studies, the efficacy of healthcare interventions can be ascertained with greater precision than through individual studies, thereby facilitating the exploration of evidence consistency of evidence across studies and the variability among them. When outcomes across multiple studies are incongruent or lack statistical significance, meta-analysis can yield statistical findings approximating the actual scenario.

### Objective

To systematically evaluate the incidence and influencing factors of PPUI.

## Methods

### PECO

P (Population): Mothers with PPUI.

E (Exposure): Various influencing factors leading to postpartum urinary incontinence in childbirth.

C (Control): Mothers without PPUI.

O (Outcome): Influencing factors and incidence of PPUI.

### Eligibility criteria

The stipulations for eligible participants were delineated as follows: (a) the presence of unambiguous diagnostic benchmarks for PPUI, predicated upon the ICS's diagnostic criteria: involuntary micturition triggered by physiological actions such as sneezing, laughter, coughing, or the lifting of considerable weight [2]; (b) participants were exempt from predisposing factors associated with high-risk pregnancies, such as advanced age, hypertension, diabetes, and other comorbidities. The principal outcome under scrutiny was the epidemiological incidence of PPUI and its corresponding determinants. Secondary outcomes were deliberately omitted from consideration. Studies disseminated in languages other than English and Chinese, systematic reviews and meta-analyses, as well as articles for which the full-text was inaccessible, were excluded.

### Search strategy

The repository (www.crd.york.ac.uk/prospero) was consulted to ascertain the existence of extant systematic reviews or meta-analyses. Titles and abstracts culled from both electronic and manual searches were integrated into the EndNote X9.1 reference management software. Reference compilations of all the considered articles were meticulously examined for potential additional studies.

Chinese and English scholarly corpus pertaining to variables influencing maternal PPUI was procured through databases such as PubMed, Embase, Cochrane Library, Web of Science, China Knowledge Network (CNKI), Vipers (VIP), Wan Fang, and Sinomed (CBM). The search temporal parameters spanned from the inception of each database to February 2023. Employed search terms included: "urinary incontinence / incontinence / incontinence of urine" AND "postpartum / delivery / pregnancy delivery / pregnancy" AND "impact factors / risk factors / relation". For a comprehensive search algorithm, refer to Table 1.

**Table 1:** Search strategy

Databases	Search strategy
**PubMed**	#1 urinary incontinence OR incontinence OR incontinence of urine
	#2 postpartum OR delivery OR pregnancy delivery OR pregnancy
	#3 impact factors OR risk factors OR relation
	#4 #1 AND #2 AND #3
	Filters applied: Full text, Humans, Chinese, English, Female
**Embase**	#1 postpartum
	#2 urinary AND incontinence
	#3 #1 AND #2
	Filters applied: Humans, Chinese, English, Female
**Web of Science**	#1 urinary incontinence
	#2 postpartum OR delivery OR pregnancy delivery OR pregnancy
	#3 impact factors OR risk factors OR relation
	#4 #1 AND #2 AND #3
	Filters applied: English
**Cochrane Library**	#1 urinary incontinence OR incontinence OR incontinence of urine
	#2 postpartum OR delivery OR pregnancy delivery OR pregnancy
	#3 impact factors OR risk factors OR relation
	#4 #1 AND #2 AND #3
	Filters applied: Chinese, English
**CNKI/VIP/CBM/Wan Fang**	#1产后 + 分娩 + 妊娠 + 产妇 + 孕产妇 + 怀孕 + 产褥 + 生产 + 产褥期
	#2失禁 + 尿失禁 + 漏尿
	#3影响因素 + 危险因素 + 关联
	#4 #1 AND #2 AND #3

### Study selection

Each title and abstract was scrutinized by two autonomous reviewers utilizing a standardized instrument [15]. Full-text articles were similarly assessed by two independent reviewers based on the following inclusion criteria: (a) Study subjects: females with unambiguous diagnostic benchmarks for PPUI and formally diagnosed with PPUI; (b) Study thematic focus: variables affecting PPUI were incorporated, and the original manuscript furnished odds ratios (OR) and 95% Confidence Intervals (CI); (c) Study methodology: cross-sectional studies, cohort studies, case–control studies; and (d) The linguistic medium of the literature was either Chinese or English. Discrepancies encountered during the screening of titles, abstracts, and full texts were amended through consultative dialogue with a third reviewer until unanimity was achieved.

### Quality assessment

The appraisal of scholarly quality was conducted independently by two investigators, and the outcomes were cross-verified. In cases of discord, a third investigator was enlisted for arbitration. Cross-sectional studies were evaluated for quality utilizing the parameters stipulated by the American Agency for Healthcare Research and Quality [16] (AHRQ). The evaluation framework comprised 11 items, with an aggregate score ranging from 8 to 11 denoting high quality, 4 to 7 signifying moderate quality, and 0 to 3 indicating low quality. Cohort and case–control studies were assessed for quality using the Newcastle–Ottawa Scale [17] (NOS). The scale encompasses nine items, with an aggregate score of 7–9 representing high quality, 5–6 denoting moderate quality, and 0–4 signifying low quality. To ensure methodological rigor, we employed the Preferred Reporting of Systematic Reviews and Meta-Analysis (PRISMA) guidelines for systematic data interrogation [18].

### Data extraction and outcome of interest

Data were culled from each study incorporated in the review using a priori-defined criteria, predicated upon the standardized JBI data extraction instrument [19]. Two authors engaged in data extraction, and comparative evaluations of the results were conducted; any dissonance was rectified through consensus. If consensus remained elusive, a third researcher was invoked to partake in the decision-making process. We initiated communication with the original authors of the qualifying studies via email or telephone for supplementary elucidation of the data. For each study, we extracted the following domains: i) Author(s) and years of publication ii) Study methodologies (cross-sectional, cohort, and case–control studies) iii) Geographic locale iv) Sample size for each cohort v) Incidence of postpartum urinary incontinence and the determinants affecting it.

### Data analysis

Data transcription was executed in Excel, and endeavors were undertaken to contact the original authors to supplement any lacunae. Baseline data, such as the first author and year of inclusion, will be tabulated in the results section.

Statistical manipulations were performed using Stata SE16.0 software. The composite effect size OR and 95% Confidence Interval (CI) were computed, and variances were deemed statistically significant if the Z-test result *P* < 0.05. Heterogeneity was gauged using I^2 in conjunction with the *P*-value of the χ^2 test: if I^2 > 50% and *P* < 0.05, it indicated substantial heterogeneity among studies, warranting the selection of a random-effects model for subsequent analytical dissection; conversely, a fixed-effects model was employed. Sensitivity examinations of the included studies were conducted by excising individual studies or modifying the analytical framework, followed by an assessment of the resultant stability of the study findings. The Egger test was deployed for ascertaining publication bias.

## Results

### Review process

**Fig. 1:**
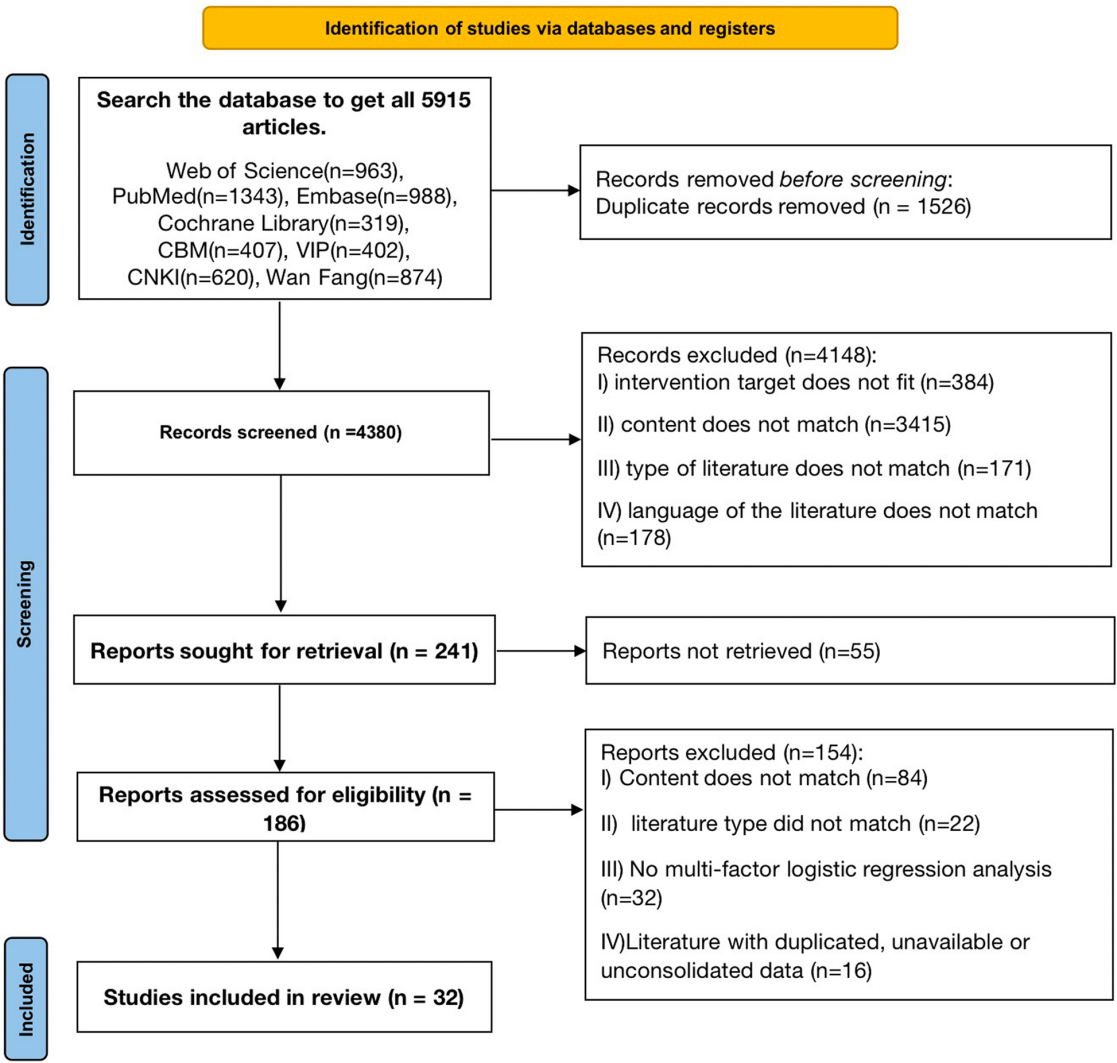
PRISMA Flow chart of search and study inclusion process

A total of 5,915 manuscripts were acquired, 1,526 duplicates were omitted, 241 manuscripts were selected during the initial screening phase by examining titles and abstracts, and ultimately 32 manuscripts were incorporated upon thorough perusal of the full texts (Fig. 1).

### Characteristics of included studies

The 32 manuscripts encompassed in this study comprised 26 manuscripts in Chinese and 6 in English. Among these, there were 2 case–control studies and 2 cohort studies, along with 28 cross-sectional studies. An aggregate of 28,303 patients were enlisted, and 6,047 patients manifested postpartum urinary incontinence. A total of 28 cross-sectional studies employed the AHRQ score, yielding evaluation results of 21 manuscripts of high quality and 7 manuscripts of moderate quality; the residual 4 manuscripts utilized the NOS score, with evaluation results indicating 1 manuscript of high quality and 3 manuscripts of moderate quality (See Table 2).

**Table 2:** Basic characteristics and quality evaluation of the included literature

Author	Years	Countries	Type of research	follow-up time (mouth)	Types of UI	Sample size (Cases)	Number of urinary incontinences (Cases)	Incidence of urinary incontinence (%)	Influencing factors^a^	Quality evaluation
Yang X [20]	2004	China	Cross-sectional studies	12	ALL	548	167	30.47	1、4	AHRQ score = 5
Yan MQ [21]	2016	China	Cross-sectional studies	/	ALL	237	103	43.46	1、2、3、9	AHRQ score = 6
Wu LY [22]	2015	China	Cross-sectional studies	1.5–2	SUI	94	19	20.21	1、6、9、10	AHRQ score = 7
Xiong YY [23]	2021	China	Cross-sectional studies	1.5–2	SUI	188	83	44.15	1、7	AHRQ score = 7
Wen L [24]	2014	China	Cross-sectional studies	6	ALL	2426	154	6.35	1、6、8、11	AHRQ score = 7
Leroy, LD [25]	2016	Brazil	Cross-sectional studies	/	ALL	344	77	22.38	6	AHRQ score = 7
Wang SJ [26]	2022	China	Cross-sectional studies	/	SUI	449	65	14.48	1、4、5	AHRQ score = 8
Di HY [27]	2021	China	Cross-sectional studies	/	SUI	258	39	15.12	2、4、6、11	AHRQ score = 8
Huang XF [28]	2016	China	Cross-sectional studies	1.5–2	ALL	1340	345	25.75	1、6	AHRQ score = 8
Cheng H [29]	2016	China	Cross-sectional studies	1.5–2	SUI	912	360	39.47	1	AHRQ score = 8
Yu J [30]	2022	China	Cross-sectional studies	/	SUI	1600	336	21.00	1、2、4、7、12	AHRQ score = 8
He H [31]	2016	China	Case–control study	1.5	SUI	200	100	/	1、4、6、8	NOS score = 6
Guo Zl [32]	2018	China	Cohort Studies	2	SUI	114	12	10.53	1、6、9、10	NOS score = 6
Wang Q [8]	2019	China	Cross-sectional studies	1.5–3	SUI	1027	303	29.50	1、2、3、7	AHRQ score = 8
Xiang JC [33]	2020	China	Cross-sectional studies	1.5–3.5	SUI	684	245	35.82	1、4、12	AHRQ score = 8
Wang D [34]	2018	China	Cross-sectional studies	1–1.5	SUI	90	18	20.00	1、6、9、10	AHRQ score = 8
Gao JJ [35]	2021	China	Cross-sectional studies	/	SUI	612	196	32.03	1、7	AHRQ score = 8
Liu J [36]	2015	China	Cross-sectional studies	/	SUI	79	41	51.90	3、4	AHRQ score = 8
Zeng JH [37]	2017	China	Cross-sectional studies	1.5–2.5	SUI	2163	836	38.65	2、3	AHRQ score = 8
Svare J [38]	2014	Denmark	Cross-sectional studies	12	ALL	745	172	23.09	6、9	AHRQ score = 8
Chen Q [39]	2013	China	Cross-sectional studies	1.5–2	SUI	82	13	15.85	1、4、6	AHRQ score = 8
Cheng, H [40]	2022	China	Cross-sectional studies	1.5	SUI	360	90	25.00	1、3	AHRQ score = 8
Chang, S.D [41]	2023	China	Cross-sectional studies	12	SUI	303	50	16.50	2、9	AHRQ score = 8
Xiong YY [42]	2022	China	Cross-sectional studies	/	SUI	500	199	39.80	1、3	AHRQ score = 9
Wan L [43]	2021	China	Case–control study	1.5	SUI	142	70	49.30	1、4、7	NOS score = 6
Wang XY [44]	2019	China	Cross-sectional studies	1.5–2	SUI	141	81	57.45	4、9	AHRQ score = 9
Zhang YY [45]	2021	China	Cross-sectional studies	1.5–2	SUI	811	71	8.75	1、2、5、9、12	AHRQ score = 9
Yang XE [46]	2020	China	Cross-sectional studies	1.5–2	SUI	475	55	11.58	1、3、5、7、8	AHRQ score = 9
Zhao X [47]	2019	China	Cross-sectional studies	1.5–2	SUI	940	92	9.79	1、3、4、7、8、9	AHRQ score = 9
Liang Y [48]	2020	China	Cross-sectional studies	1.5–2	ALL	717	120	16.74	1、2	AHRQ score = 9
Shi W [49]	2019	China	Cross-sectional studies	1.5	ALL	9550	1483	15.53	2、3、4、6、7、9、11	AHRQ score = 9
Zhong, R [50]	2022	China	Cohort Studies	12	SUI	172	52	30.23	2、6	NOS score = 7

^a^Influencing factors: 1. mode of delivery; 2. age; 3. multiparity (number of deliveries ≥ 2); 4. neonatal weight; 5. perineal dystonia; 6.antecedents of urological incontinence- related pathology; 7. maternal pre- conception BMI; 8. postpartum functional training of the pelvic floor muscles; 9. perineal laceration; 10. instrumental parturition; 11.historical pelvic surgical procedures; 12. protracted second stage of labor

### Weighted mean difference of PPUI

**Fig. 2:**
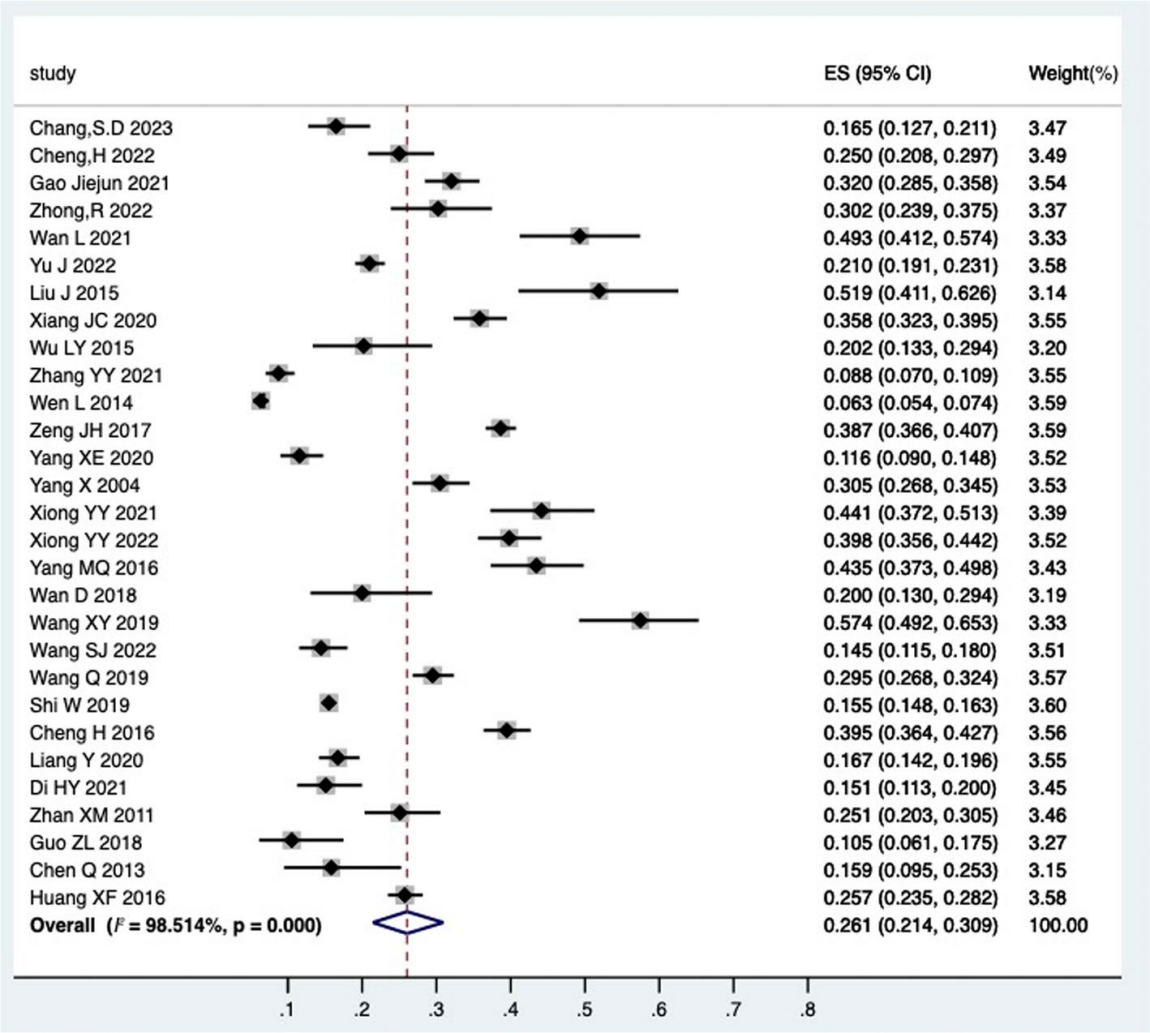
Forest plot of the incidence of postpartum urinary incontinence

**Fig. 3:**
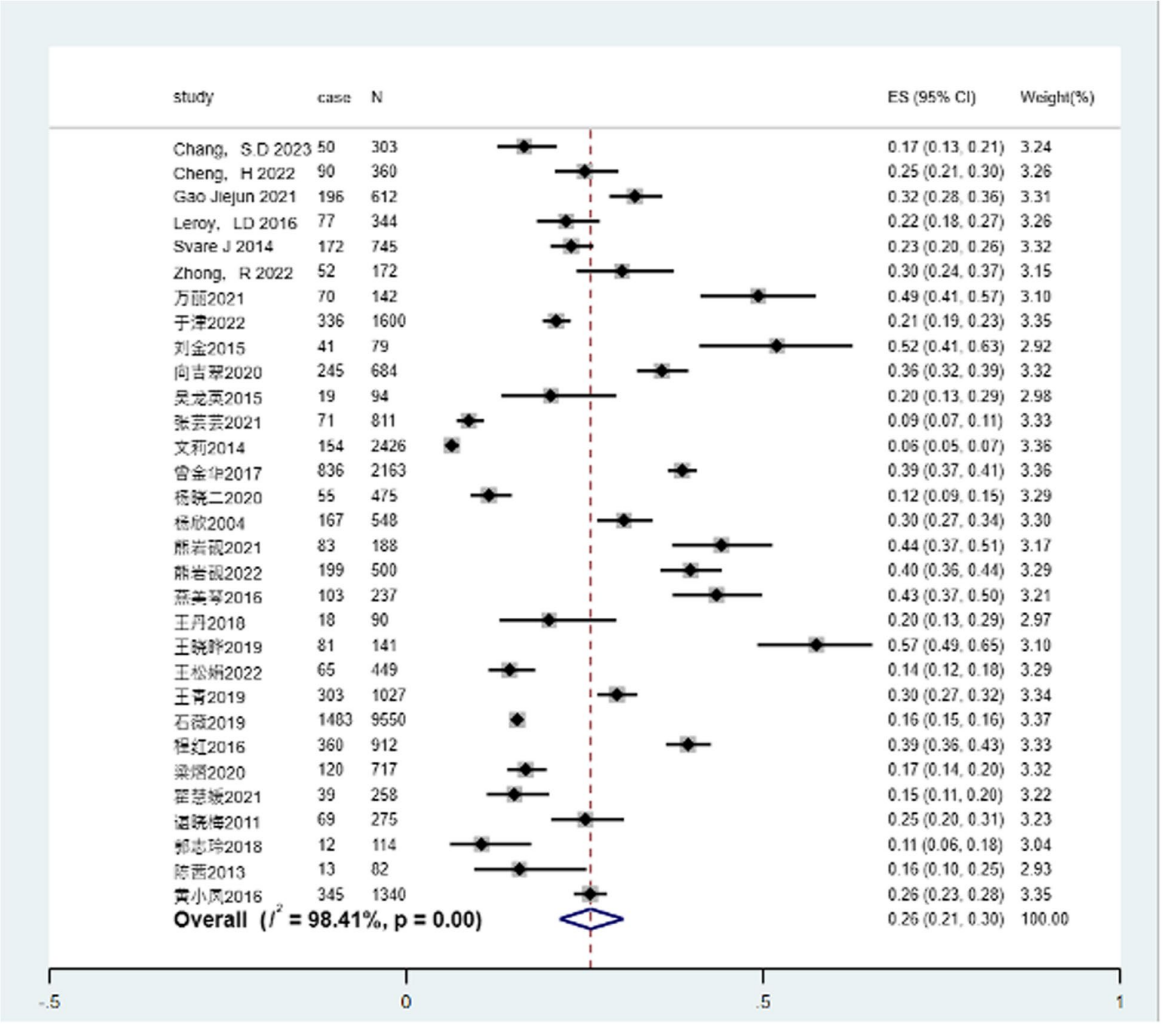
Forest plot of the incidence of postpartum urinary incontinence in China

A heterogeneity test was administered on the 31 retained manuscripts (one manuscript exhibited no incidence of PPUI and was consequently excluded), and the results revealed significant heterogeneity between studies (I^2 = 98.41%, *P* < 0.05), thus a random-effects model was designated for analysis. The consequent meta-analysis demonstrated that the incidence of PPUI was 26% [95% CI (21%—30%)] (Fig. 2). Further analysis indicated that the incidence of PPUI in China was 26% [95% CI (21%—31%)] (Fig. 3). This is congruent with prior documentations of the incidence of PPUI [51].

### Sensitivity analysis of postpartum incontinence

**Fig. 4:**
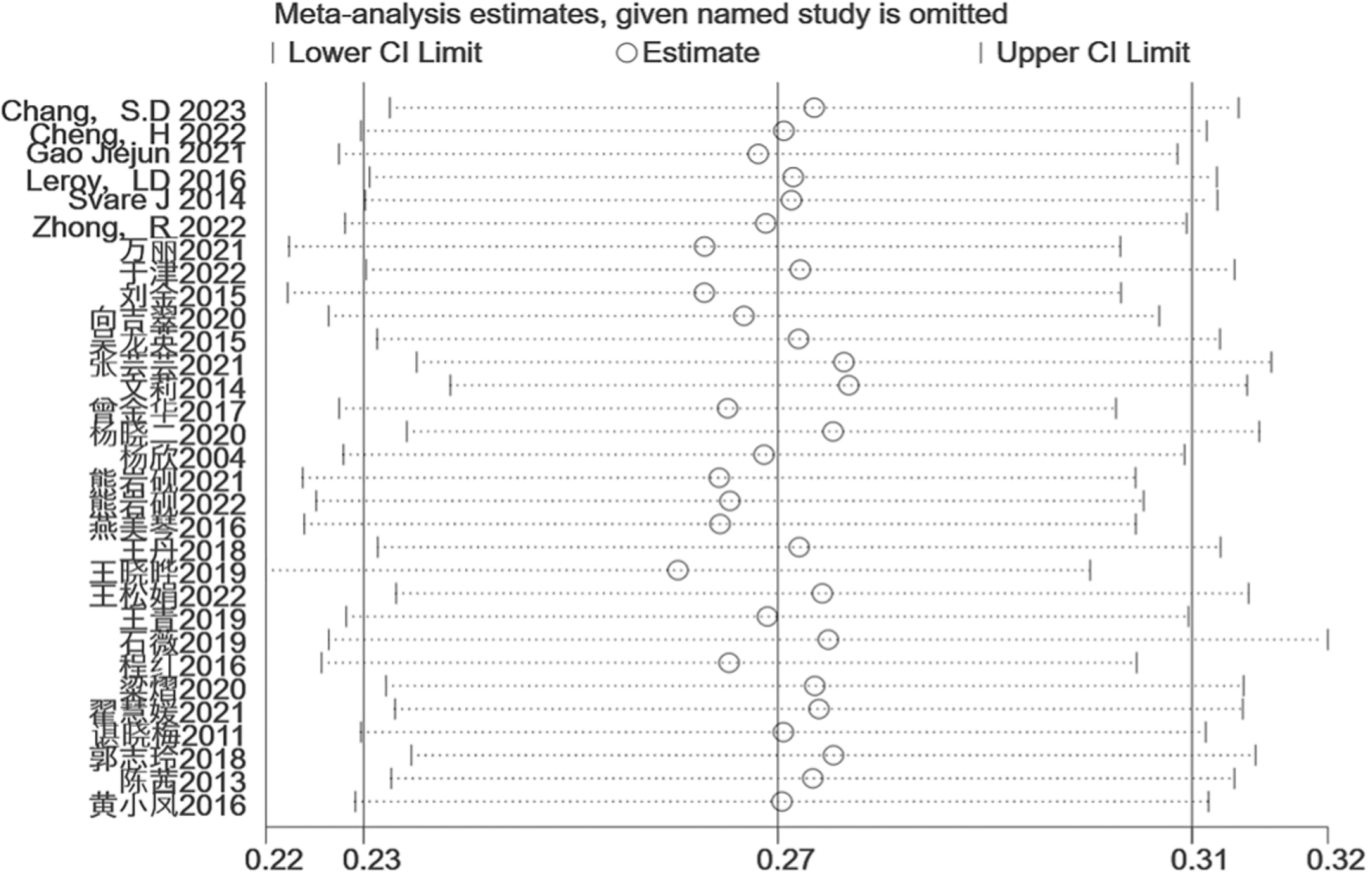
Sensitivity analysis of postpartum urinary incontinence

Upon conducting a sensitivity analysis to exclude individual studies sequentially, the incidence of composite debilitation remained invariant, signifying that the results were robust and reliable (Fig. 4).

### Assessment of publication bias of postpartum incontinence

**Fig. 5:**
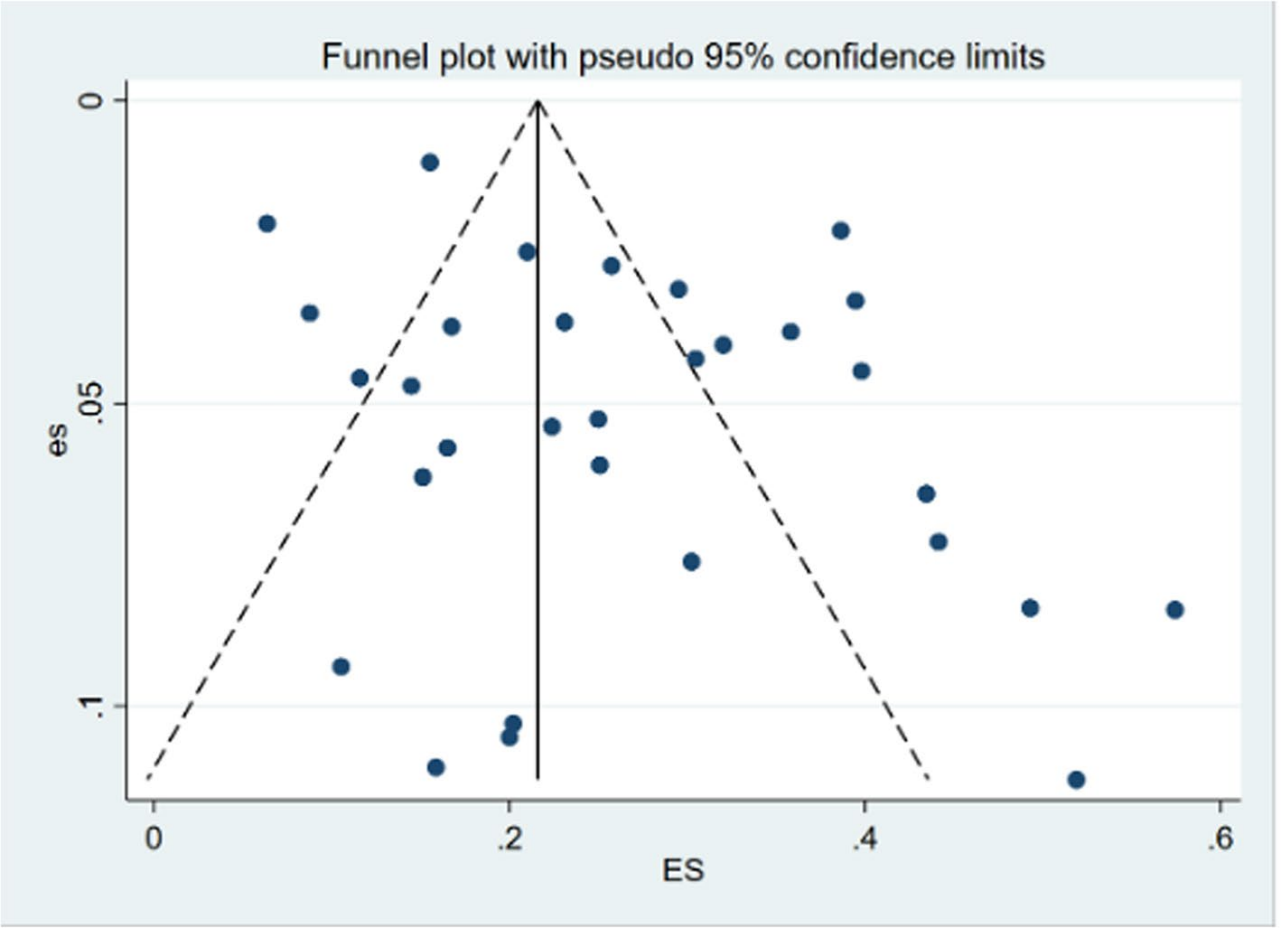
Funnel plot of the incidence of postpartum urinary incontinence

The funnel plot, coupled with Egger's test (t = 2.28, *P* = 0.023), for the aggregate incidence of postpartum incontinence unveiled some degree of publication bias in this investigation (Fig. 5).

### A meta-analysis of factors influencing postpartum urinary incontinence

Parity, advanced maternal age (≥ 35 years), multiparity (number of deliveries ≥ 2), elevated neonatal weight, perineal deviation, antecedent history of urinary incontinence pathology, augmented pre-pregnancy BMI, perineal laceration, instrumental parturition, pelvic surgical theater, and extended duration of the second stage of labor were all identified as salient risk determinants for maternal postpartum urinary incontinence (*p* < 0.05). Conversely, cesarean section emerged as a protective determinant against maternal postpartum urinary incontinence (*p* < 0.001), as delineated in Table 3.

**Table 3:** Meta-analysis of factors influencing postpartum urinary incontinence

Influencing factors	Number of included studies	Heterogeneity test			Meta-analysis results	
		Q	*P*	*I*^2^	OR(95%CI)	*P*
Cesarean delivery	23 [20–24, 26, 28–35, 39, 40, 42, 43, 45–48]	20.14	0.001	75.20%	0.416 (0.282–0.612)	< 0.001
Vaginal delivery	23 [20–24, 26, 28–35, 39, 40, 42, 43, 45–48]	209.5	< 0.001	92.40%	3.085 (2.163–4.398)	< 0.001
Age (≥ 35 years old)	10 [8, 21, 27, 30, 37, 41, 45, 48–50]	104.56	< 0.001	91.40%	1.493 (1.274–1.750)	< 0.001
Multiparity (number of deliveries ≥ 2)	9 [8, 21, 36, 37, 40, 42, 46, 47, 49]	20.43	0.009	60.80%	1.736 (1.357–2.222)	< 0.001
Neonatal weight > 4 kg	12 [20, 26, 27, 30, 31, 33, 36, 39, 43, 44, 47, 49]	27.81	0.003	60.50%	2.142 (1.688–2.717)	< 0.001
Lateral episiotomy	3 [26, 45, 46]	1.95	0.377	0.00%	1.811 (1.359–2.413)	< 0.001
Antecedent history of UI pathology	12 [22, 24, 25, 27, 28, 31, 32, 34, 38, 39, 49, 50]	102.58	< 0.001	89.30%	3.651 (2.488–5.358)	< 0.001
Augmented pre-pregnancy BMI ≥ 24 kg/m^2^	8 [8, 23, 30, 35, 43, 46, 47, 49]	47.85	< 0.001	85.40%	1.657 (1.294–2.123)	< 0.001
Postpartum functional exercises for pelvic floor muscles	4 [24, 31, 46, 47]	11.92	0.008	74.80%	1.066 (0.519–2.187)	0.862
Perineal deviation	10 [21, 22, 32, 34, 38, 41, 44, 45, 47, 49]	63.91	< 0.001	85.90%	1.664 (1.277–2.168)	< 0.001
Instrumental parturition	3 [22, 32, 34]	5.07	0.079	60.50%	1.791 (1.133–2.833)	0.013
Pelvic surgical theater	3 [24, 27, 29]	5.1	0.078	60.80%	1.379 (1.003–1.897)	0.048
Prolonged second stage of labor	3 [30, 33, 45]	3.97	0.138	49.60%	2.413 (1.917–3.038)	< 0.001

### Sensitivity analysis and publication bias of factors influencing postpartum urinary incontinence

A sensitivity analysis was implemented for the aforementioned contributory variables, and the aggregate effect magnitudes were ascertained independently by modifying the two effect models. The findings indicated that the aggregate effect magnitudes under the two models remained statistically invariant, corroborating that the results were robust and unassailable, as manifested in Table 4. Publication bias assessments were executed solely for the contributory variables for which ≥ 10 manuscripts were incorporated, and the corresponding test results are enumerated in Table 5.

**Table 4:** Sensitivity analysis of influencing factors

Influencing factors	Fixed effects model OR(95%CI)	Random effects model OR (95%CI)
Cesarean delivery	0.450 (0.378–0.535)	0.416 (0.282–0.613)
Vaginal delivery	1.024 (1.016–1.032)	3.085 (2.163–4.398)
Age (≥ 35 years old)	1.085 (1.063–1.108)	1.493 (1.274–1.750)
Multiparity (number of deliveries ≥ 2)	1.497 (1.315–1.703)	1.736 (1.357–2.222)
Neonatal weight > 4 kg	1.824 (1.604–2.074)	2.142 (1.688–2.717)
Lateral episiotomy	1.811 (1.359–2.413)	1.811 (1.359–2.413)
Antecedent history of UI pathology	2.215 (2.141–2.290)	3.651 (2.488–5.358)
Augmented pre-pregnancy BMI ≥ 24 kg/m^2^	1.099 (1.053–1.146)	1.657 (1.294–2.123)
Perineal deviation	1.446 (1.335–1.566)	1.664 (1.277–2.168)
Instrumental parturition	1.370 (1.349–1.390)	1.791 (1.133–2.832)
Pelvic surgical theater	1.294 (1.097–1.526)	1.379 (1.003–1.897)
Prolonged second stage of labor	2.449 (1.761–3.407)	2.413 (1.917–3.407)

**Table 5:** Egger test for influencing factors

Influencing factors	t	*P*
Vaginal delivery	16.02	< 0.001
Age (≥ 35 years old)	4.41	0.002
Neonatal weight > 4 kg	4.91	0.001
Antecedent history of UI pathology	2.62	0.025
Perineal deviation	0.99	0.350

## Discussion

The current meta-analysis endeavored to scrutinize the relationship in a comprehensive and methodical manner between diverse predisposing factors and PPUI. We aspired to furnish aggregated empirical evidence concerning predictive variables implicated in the onset of postpartum SUI. The outcomes of the meta-analysis revealed that the incidence of maternal PPUI was 26% [95% CI (21%-30%)], which conforms to the previously documented incidence range of PPUI spanning 10% to 63% across various geographical locales [13].

Gestation and parturition represent distinctive epochs in a woman’s life, while simultaneously serving as potent etiological contributors to pelvic floor dysfunctional maladies [44]. When juxtaposed with prior meta-analyses authored by Kai Wang [14] et al. and Tähtinen [52] et al., consensus prevails that both vaginal parturition and advancing age constitute salient risk factors for postpartum urinary incontinence. It remains unequivocal that vaginal delivery represents the most paramount risk determinant for postpartum urinary incontinence. As gestation progresses, the expanding gravid uterus exerts incremental pressure upon the bladder and surrounding anatomical structures. Concomitant with marked hormonal fluctuations, the pelvic floor musculature and supportive tissues manifest diminished tensile strength, culminating in compromised sphincter control [45], thereby amplifying the prevalence of PPUI. Both cesarean and euploid deliveries induce substantial perturbations in maternal pelvic floor integrity. In the context of euploid parturition, such structural alterations are exacerbated during the fetal emergence phase. Conversely, during cesarean delivery, the neonate abstains from exerting pressure on pelvic floor structures, thus mitigating the likelihood of PPUI development. While euploid delivery does augment PPUI prevalence, elective cesarean delivery should not be adopted solely on this premise. Rather, preemptive interventions, such as the early initiation of Kegel exercises [53], are advocated for those opting for vaginal parturition. Emerging data corroborate that a growing proportion of women are undergoing their inaugural deliveries at an advanced maternal age [54]. Given the expanding demographic of women classified within this risk stratum, the clinical pertinence of maternal age as a contributory factor to SUI amplifies [55]. Typically, a maternal age of 35 years or greater is designated as advanced. The field of biomedicine categorizes gestations among such women as high-risk endeavors [56]. Elevated age at parturition escalates the probability of multifaceted gestational complications. Concomitant with aging, pelvic floor musculature exhibits varied degrees of laxity, and the diminishing physiological vigor in older women translates into suboptimal postpartum recuperation, thereby elevating PPUI risk. Consequently, women should be exhorted to conceive at medically advisable ages.

In alignment with Kai Wang’s [14] investigation, we postulate that lateral incisions, instrumental deliveries, and prior medical antecedents pertinent to UI constitute risk factors for PPUI. Study [57] demonstrated that lateral episiotomy facilitates the expeditious parturition of the fetus and concurrently serves to mitigate the incidence of severe perineal lacerations. Conjoined with the unproblematic and orderly cicatrization of the lateral incision, it is conjectured that the lateral episiotomy exerts a specific prophylactic influence on pelvic floor musculature. However, contemporary international scholarship [58] indicates that lateral episiotomy lacks prophylactic efficacy for the pelvic floor musculature and may precipitate pelvic floor disorders such as stress urinary incontinence. Lateral episiotomy and perineal lacerations can compromise perineal nerves and musculature, undermining the structural integrity of pelvic floor nerves and muscles, which subsequently impinges upon the contractile functionality of the pelvic floor muscle groups and the mechanisms regulating urinary continence [59]. Thus, it is imperative to expeditiously reinstate the structural and functional integrity of the pelvic floor musculature in women who have experienced perineal lacerations or episiotomies.

Sultan’s study [60] additionally substantiates that stress urinary incontinence correlates with pelvic floor tissue compromise and the prolongation of the second stage of labor, as well as with fetal cranial dimensions and mass. These correlations resonate with our extant research insights.

Moreover, in distinction to prevailing studies, our review ascertains that the number of deliveries also serves as a risk factor for postpartum urinary incontinence. Each gestational and parturitional event inflicts heterogeneous magnitudes of detriment to pelvic floor functionality. This deleterious impact amplifies in correspondence with the multiplicity of deliveries. For women experiencing multiple gestations and parturitions, prophylactic interventions against PPUI should be implemented.

Beyond the obstetric variables delineated heretofore, we contend that non-obstetric variables likewise contribute substantively to the genesis of postpartum urinary incontinence. Meta-analysis reveals that maternal pre-pregnancy BMI indices and the history of pelvic surgical interventions emerge as additional risk determinants for postpartum urinary incontinence. These findings imply a potential preventive strategy against PPUI through regulation of maternal pre-gestational BMI parameters. Concomitantly, in women manifesting a historical trajectory of pelvic surgical interventions, pre-existing pelvic floor functionality stands inherently compromised. Such individuals warrant educational guidance during their gestational phase, aimed at preliminary prevention of PPUI.

## Conclusion

PPUI exerts a detrimental impact on the physical and psychological well-being of women; thus, prophylactic and therapeutic interventions for postpartum incontinence should be initiated expeditiously to ameliorate the prevalence of PPUI and enhance postpartum maternal quality of life. Numerous variables influence postpartum urinary incontinence; healthcare providers must ascertain these high-risk determinants of PPUI in a timely manner and proffer targeted preventive guidance to mitigate the incidence fundamentally.

### Limitation of this review

Firstly, the study omitted scholarly works comprising fewer than two studies on the same influencing factor or possessing data that were not amenable to synthesis or utilization, potentially introducing selection bias. Secondly, significant heterogeneity was manifest in the meta-analysis concerning the incidence of postpartum urinary incontinence, and the genesis of this heterogeneity could be attributed to the temporal and geographical contexts of the culled literature, thereby impacting the generalizability of the findings. Future research could focus on the incidence of postpartum urinary incontinence and its influential variables among Chinese mothers. Lastly, the restricted sample size constrained this study, with some influencing factors being represented in an insufficient corpus of literature, thus precluding the assessment of publication bias. Future research endeavors could benefit from large-scale, multicentric prospective cohort studies to further corroborate these preliminary findings.

## Data Availability

The datasets supporting the conclusions of this article are included within the article.

## Abbreviations

PPUI: Postpartum Urinary Incontinence
BMI: Body Mass Index
ICS: The International Continence Society
SUI: Stress Urinary Incontinence
UUI: Urgency Urinary Incontinence
MUI: Mixed Urinary Incontinence

## References

[1] Diz-Teixeira P, Alonso-Calvete A, Justo-Cousiño LA (2023). Update on physiotherapy in postpartum urinary incontinence. A systematic review. Arch Esp Urol.

[2] Abrams P, Cardozo L, Fall M (2003). The standardisation of terminology in lower urinary tract function: report from the standardisation sub-committee of the International Continence Society. Urology.

[3] Stadnicka G, Łepecka-Klusek C, Pilewska-Kozak A (2015). Psychosocial problems of women with stress urinary incontinence. Ann Agric Environ Med.

[4] Nam JY, Park EC, Cho E (2021). Does urinary incontinence and mode of delivery affect postpartum depression? A nationwide population-based cohort study in Korea. Int J Environ Res Public Health.

[5] Hullfish KL, Fenner DE, Sorser SA (2007). Postpartum depression, urge urinary incontinence, and overactive bladder syndrome: is there an association?. Int Urogynecol J Pelvic Floor Dysfunct.

[6] Suar G, Cevik F, Simal Yavuz N (2023). Urinary incontinence in the postpartum 1-year period: Its prevalence and effect on psychosocial status of women. Low Urin Tract Symptoms..

[7] Hage-Fransen MAH, Wiezer M, Otto A (2021). Pregnancy- and obstetric-related risk factors for urinary incontinence, fecal incontinence, or pelvic organ prolapse later in life: a systematic review and meta-analysis. Acta Obstet Gynecol Scand.

[8] Wang Q, Yu XJ, Chen GM (2019). Risk factors for urinary incontinence after delivery. Chin J Clin Obstet Gynecol.

[9] Feng Y, Sun K, Chen XY (2019). Clinical study of the factors influencing the occurrence of postpartum stress urinary incontinence. Electronic J Pract Gynecol Endocrinol.

[10] Sangsawang B, Sangsawang N (2013). Stress urinary incontinence in pregnant women: a review of prevalence, pathophysiology, and treatment. Int Urogynecol J.

[11] Chang SR, Lin WA, Chang TC (2021). Risk factors for stress and urge urinary incontinence during pregnancy and the first year postpartum: a prospective longitudinal study. Int Urogynecol J.

[12] Yuan JY, Chen WR, Zhao JP (2016). Retrospective analysis of the incindence and influencing factors of 190 postpartum women with post-partum ueinary incontinence. Chin Med Record.

[13] Moossdorff-Steinhauser HFA, Berghmans BCM, Spaanderman MEA (2021). Prevalence, incidence and bothersomeness of urinary incontinence between 6 weeks and 1 year post-partum: a systematic review and meta-analysis. Int Urogynecol J.

[14] Wang K, Xu X, Jia G (2020). Risk factors for postpartum stress urinary incontinence: a systematic review and meta-analysis. Reprod Sci.

[15] Porritt K, Gomersall J, Lockwood C (2014). JBI's Systematic Reviews: Study selection and critical appraisal. Am J Nurs.

[16] Lusk SL (2000). American academy of nursing senior scholar at the agency for healthcare research and quality. Nurs Outlook.

[17] Stang A (2010). Critical evaluation of the Newcastle-Ottawa scale for the assessment of the quality of nonrandomized studies in meta-analyses. Eur J Epidemiol.

[18] Page MJ, McKenzie JE, Bossuyt PM (2021). The PRISMA 2020 statement: an updated guideline for reporting systematic reviews. BMJ.

[19] Moola S, Munn Z, Sears K (2015). Conducting systematic reviews of association (etiology): the Joanna Briggs Institute's approach. Int J Evid Based Healthc.

[20] Yang X, Zheng H, Liao QP (2004). Mode of delivery on urinary incontinence. Chin J Obstet Gynecol.

[21] Yang MQ, Wang LM, Wang J (2016). Study on the correlation between labor factors and urinary incontinence. Mater Child Health Care China.

[22] Wu LY, Wei WW, Tao L (2015). The influence of pregnancy, delivery and obstetric factors on postpartum stress urinary incontinence. Anhui Med Pharmaceutical J.

[23] Xiong YY (2021). Analysis of risk factors for early postpartum stress urinary incontinence in postpartum women. Med J Chin Peoples Health.

[24] Wen L, Zhang B, Yi NH (2014). Analysis of the incidence and influencing factors of urinary incontinence in primiparous women. Lab Med Clin.

[25] Leroy LA, Lúcio A, Lopes MH (2016). Risk factors for postpartum urinary incontinence. Revista Enferm USP.

[26] Wang SJ, Guo YS, Lin F (2022). Study on the correlation between fetal weight and postpartum stress urinary incontinence of primipara. Chin J Fam Plan Gynecotokol.

[27] Di HY (2021). Study on clinical risk factors of early postpartum stress urinary incontinence in postpartum women. Syst Med.

[28] Huang XF, Wei HW, Qiu XX (2016). A study on the related factors of primiparas’urinary incontinence in Guangxi Zhuang autonomous Region. J Chengdu Med Coll.

[29] Cheng H (2016). The influencing factor analysis of stress urinary incontinence in 912 postpartum women in Fuyang. J Bengbu Med Coll.

[30] Yu J, Xie J (2022). Risk factors of postpartum stress urinary incontinence and construction of a prediction model in primiparas. J Tongji Univ (Medical Science).

[31] He H (2016). Risk factors for stress urinary incontinence in postpartum women. Mater Child Health Care China.

[32] Guo ZL (2018). The impact of obstetric related factors and delivery methods on stress urinary incontinence. Henan Med Res.

[33] Xiang JC (2020). The influencing factors and predictive models of postpartum stress urinary incontinence. J Med Theory Pract.

[34] Wang D (2018). The effect of different delivery methods on postpartum stress urinary incontinence. J Shandong Med Coll.

[35] Jiejun G, Xinru L, Yan Z (2021). Risk factors of postpartum stress urinary incontinence in primiparas: What should we care. Medicine.

[36] Liu J, Zhou JL, Jin ZY (2015). Analysis of factors associated with the occurrence of stress urinary incontinence at full term of pregnancy and ultrasound parameters. Mater Child Health Care of China.

[37] Zeng JH, Wang GY, Lin SH (2017). Epidemiological investigation on female stress urinary incontinence at 42 days after delivery. Mater Child Health Care China.

[38] Svare JA, Hansen BB, Lose G (2014). Risk factors for urinary incontinence 1 year after the first vaginal delivery in a cohort of primiparous Danish women. Int Urogynecol J Pelvic Floor Dysfunct.

[39] Chen Q, Wang XZ, Li QM (2013). Effect of delivery way and collagen metabolism on postpartum stress urinary incontinence. China Med Herald.

[40] Cheng H, Gong F, Shen Y (2022). A nomogram model predicting the risk of postpartum stress urinary incontinence in primiparas: a multicenter study. Taiwan J Obstet Gynecol.

[41] Chang SD, Hsieh WC, Chiu SYH (2023). Factors determining the persistence of prenatal stress urinary incontinence 12 months postpartum. Taiwan J Obstet Gynecol.

[42] Xiong YY (2022). Logistic regression analysis of birth factors of stress urinary incontinence. Med Forum.

[43] Wang L, Sun JY, Gu YY (2021). Observation on the therapeutic effect of pelvic floor muscle training on postpartum stress urinary incontinence and analysis of influencing factors. Military Med J Southeast China.

[44] Wang XY, Zhong RX, Wang Y (2019). The clinical risk factors of early postpartum stress urinary incontinence in multipara. Chin J Clin Obstet Gynecol.

[45] Zhang YY, He ZZ, Zuo LY (2021). Analysis of postpartum early pelvic floor function status in primiparas and its influencing factors of stress urinary incontinence. J Clin Med Pract.

[46] Yang XE, Cheng F, Wang H (2020). Risk factors of postpartum stress urinary incontinence. Pract Prev Med.

[47] Zhao X, Reng ZH (2019). Analysis of relevant factors and prevention and treatment strategies for postpartum stress urinary incontinence. Mater Child Health Care China.

[48] Liang Y, Wang Q, Li XD (2020). Status and influencing factors of urine leakage in postpartum women. J Nurs Sci.

[49] Shi W, Niu XY, Chen YY (2019). A Study on the risk factors for early postpartum urinary incontinence in Chengdu. J Sichuan Univ (Medical Sciences).

[50] Zhong RX, Zeng L, Wang XY (2022). A retrospective study of risk factors for stress urinary incontinence 1 year after delivery in multiparous women. Int Urogynecol J.

[51] Ku XX. Research on the present situation and effect factors of postpartum urinary incontinence (Thesis). Jinzhou Med Univ. 2019;13–4.

[52] Tähtinen RM, Cartwright R, Tsui JF (2016). Long-term Impact of mode of delivery on stress urinary incontinence and urgency urinary incontinence: a systematic review and meta-analysis. Eur Urol.

[53] Huang YC, Chang KV. Kegel Exercises. In: StatPearls. Treasure Island (FL): StatPearls Publishing; 2023. 32310358

[54] Huang L, Sauve R, Birkett N (2008). Maternal age and risk of stillbirth: a systematic review. CMAJ.

[55] Hijaz A, Sadeghi Z, Byrne L (2012). Advanced maternal age as a risk factor for stress urinary incontinence: a review of the literature. Int Urogynecol J.

[56] Mann ES, Berkowitz D (2023). The biomedical subjectification of women of advanced maternal age: reproductive risk, privilege, and the illusion of control. J Health Soc Behav.

[57] Thomaz RP, Colla C, Darski C (2018). Influence of pelvic floor muscle fatigue on stress urinary incontinence: a systematic review. Int Urogynecol J.

[58] Akin Y, Young M, Elmussareh M (2018). The novel and minimally invasive treatment modalities for female pelvic floor muscle dysfunction Beyond the Traditional. Balkan Med J.

[59] Chmielewska D, Stania M, Kucab-Klich K (2019). Electromyographic characteristics of pelvic floor muscles in women with stress urinary incontinence following sEMG-assisted biofeedback training and Pilates exercises. PLoS One.

[60] Sultan AH, Kamm MA, Hudson CN (1994). Pudendal nerve damage during labour: prospective study before and after childbirth. Br J Obstet Gynaecol.

